# A validated LC-MS/MS assay for quantification of 24(S)-hydroxycholesterol in plasma and cerebrospinal fluid[S]

**DOI:** 10.1194/jlr.D058487

**Published:** 2015-06

**Authors:** Rohini Sidhu, Hui Jiang, Nicole Y. Farhat, Nuria Carrillo-Carrasco, Myra Woolery, Elizabeth Ottinger, Forbes D. Porter, Jean E. Schaffer, Daniel S. Ory, Xuntian Jiang

**Affiliations:** * Diabetic Cardiovascular Disease Center, Washington University School of Medicine, St. Louis, MO 63110; † Program in Developmental Endocrinology and Genetics, Eunice Kennedy Shriver National Institute of Child Health and Human Development, National Institutes of Health, Bethesda, MD 20892; ** Nursing Department, National Institutes of Health, Bethesda, MD 20892; § Therapeutics for Rare and Neglected Diseases National Center for Advancing Translational Sciences, National Institutes of Health, Rockville, MD 20850; †† Division of Preclinical Innovation, National Center for Advancing Translational Sciences, National Institutes of Health, Rockville, MD 20850

**Keywords:** two-dimensional liquid chromatography-tandem mass spectrometry, validation, Niemann-Pick C1 disease, 2-hydroxypropyl-β-cyclodextrin

## Abstract

24(S)-hydroxycholesterol [24(S)-HC] is a cholesterol metabolite that is formed almost exclusively in the brain. The concentrations of 24(S)-HC in cerebrospinal fluid (CSF) and/or plasma might be a sensitive marker of altered cholesterol metabolism in the CNS. A highly sensitive 2D-LC-MS/MS assay was developed for the quantification of 24(S)-HC in human plasma and CSF. In the development of an assay for 24(S)-HC in CSF, significant nonspecific binding of 24(S)-HC was observed and resolved with the addition of 2.5% 2-hydroxypropyl-β-cyclodextrin (HP-β-CD) into CSF samples. The sample preparation consists of liquid-liquid extraction with methyl-tert-butyl ether and derivatization with nicotinic acid. Good linearity was observed in a range from 1 to 200 ng/ml and from 0.025 to 5 ng/ml, for plasma and CSF, respectively. Acceptable precision and accuracy were obtained for concentrations over the calibration curve ranges. Stability of 24(S)-HC was reported under a variety of storage conditions. This method has been successfully applied to support a National Institutes of Health-sponsored clinical trial of HP-β-CD in Niemann-Pick type C1 patients, in which 24(S)-HC is used as a pharmacodynamic biomarker.

In the CNS, cholesterol originates almost exclusively from in situ synthesis (1), while circulating cholesterol is normally prevented from entering the CNS by the blood-brain barrier (2). As cholesterol cannot be eliminated in the CNS, and may be toxic to neurons when in excess, it is secreted from the CNS into the circulation predominantly in the form of its polar metabolite, 24(S)-hydroxycholesterol [24(S)-HC] (3). 24(S)-HC is formed almost exclusively in the brain. The enzymatic conversion of CNS cholesterol to 24(S)-HC, which readily crosses the blood-brain barrier, is the major pathway to eliminate cholesterol and maintain cholesterol homeostasis in brain tissue. The cholesterol 24-hydroxylase (CYP46A1) mediating this conversion is mainly located in neurons (4). The concentrations of 24(S)-HC in cerebrospinal fluid (CSF) and/or plasma might be a sensitive marker of increased cholesterol metabolism in the CNS. Plasma 24(S)-HC is decreased in Alzheimer’s disease, vascular dementia, multiple sclerosis, Parkinson’s disease, and Huntington’s disease, reflecting disease burden, the loss of metabolically active neurons, and the degree of structural atrophy (5–17). Increased cholesterol turnover (i.e., myelin breakdown or neurodegeneration), which occurs at an early stage in these diseases, appears to be associated with a transient increase of 24(S)-HC efflux and higher plasma or CSF 24(S)-HC concentration (18, 19).

Previously, quantification of 24(S)-HC in biological samples was included in oxysterol and steroid analysis using GC or LC coupled with MS. The measurement of total 24(S)-HC was performed after an alkaline hydrolysis of esterified sterols, and the alkaline hydrolysis step was omitted if only free or unesterified 24(S)-HC was measured. GC-MS is widely used for measurement of oxysterols, such as 24(S)-HC, due to its chromatographic resolving capacity, but this method has limitations such as less sample capacity and long GC run (>15 min) (20–22). LC-MS/MS was demonstrated as a sensitive, specific, and rapid method for the quantification of 24(S)-HC in biological samples. The atmospheric pressure chemical ionization (APCI) (23–25) and atmospheric pressure photoionization (26) allow direct, but less sensitive, analysis of 24(S)-HC without derivatization. Although 24(S)-HC can be detected as the [M+NH_4_]^+^ ion in ESI (27, 28), the sensitivity was greatly enhanced after derivatization with (2-hydrazinyl-2-oxoethyl) trimethylazanium chloride (Girard reagent) (29–33), *N*,*N*-dimethylglycine (34), picolinic acid (35), and 4-(dimethylamino)phenyl isocyanate (36). As 24(S)-HC cannot be differentiated by MS from many positional isomers, chromatographic resolution by a long LC run (≥12 min) in most methods is crucial for analysis of 24(S)-HC. While highly abundant 24(S)-HC in plasma and serum (23, 25, 27, 28, 30, 35) has been analyzed by LC-MS/MS and GC-MS (20–22), measurement of low abundant 24(S)-HC in CSF by GC-MS requires large sample volumes (37).

Here, we report a sensitive and robust LC-MS/MS method with a total run time of 7.5 min for determination of free 24(S)-HC in human plasma and CSF involving a liquid-liquid extraction and derivatization into nicotinate. The lower limits of quantification (LLOQs) were found to be 1 and 0.025 ng/ml for plasma and CSF, respectively. The validated method has been successfully applied to support a National Institutes of Health (NIH)-sponsored clinical trial of 2-hydroxypropyl-β-cyclodextrin (HP-β-CD) in Niemann-Pick type C1 (NPC1) patients, in which 24(S)-HC was explored as a pharmacodynamic biomarker (38).

## MATERIALS AND METHODS

### Chemicals and reagents

24(S)-HC was obtained from Avanti Polar Lipids (Alabaster, AL). 25,26,26,26,27,27,27-[^2^H_7_]24(R/S)-hydroxycholesterol (D7-24-HC) was obtained from Medical Isotopes, Inc. (Pelham, NH). Nicotinic acid, *N*,*N*′-diisopropylcarbodiimide, 4-(dimethylamino)pyridine, formic acid, ammonium acetate, methyl tert-butyl ether, chloroform, and BSA were obtained from Sigma-Aldrich (St. Louis, MO). All HPLC solvents (methanol and acetonitrile) were HPLC grade and were purchased from EMD Chemicals (Gibbstown, NJ). Milli-Q ultrapure water was prepared in-house with a Milli-Q Integral Water Purification System (Billerica, MA). The HP-β-CD was purchased from Roquette (Lestrem, Cedex, France). Pooled control human plasma (K_2_EDTA), human CSF, six lots of individual human plasma, and six lots of individual human CSF were purchased from BioChemed Services (Winchester, VA). The HP-β-CD was added to CSF to reach a final concentration of 2.5%.

### Stock solution preparation

All the stock solutions (1 mg/ml) were prepared in methanol. A working solution containing 10 μg/ml of 24(S)-HC was prepared by the dilution of the stock solution with methanol. The internal standard working solutions for plasma (50 ng/ml of D7-24-HC) and CSF (5 ng/ml of D7-24-HC) were prepared in methanol-water (1:1).

### Standard curves

Because of the endogenous presence of 24(S)-HC in human plasma and CSF, aqueous solutions of 5% BSA and 2.5% HP-β-CD were used to prepare the calibration standards for plasma and CSF, respectively. Calibration curves were prepared by spiking the 24(S)-HC working solution into 5% BSA and 2.5% HP-β-CD solutions, and preparing serial dilutions that yielded eight calibration standards (1, 2, 5, 10, 20, 50, 100, and 200 ng/ml for plasma assay; 0.025, 0.05, 0.1, 0.25, 0.5, 1, 2.5, and 5 ng/ml for CSF assay). Five percent BSA and 2.5% HP-β-CD solutions served as blanks. The same calibration standards in plasma and CSF were also prepared and used to assess responsiveness in different matrixes, which was evaluated by parallelism between standard curves prepared in biological matrix (plasma and CSF) and surrogate matrix (5% BSA and 2.5% HP-β-CD in water).

### Quality control samples

The pooled-plasma and CSF samples were analyzed to establish the mean concentration of endogenous 24(S)-HC by the LC/MS/MS method. The low quality control (LQC), middle quality control (MQC), high quality control (HQC), and dilution quality control (DQC) samples [endogenous level (+0 ng/ml), endogenous level (+75 ng/ml), endogenous level (+150 ng/ml), and endogenous level (+300 ng/ml) for human plasma assay; endogenous level (+0 ng/ml), endogenous level (+2 ng/ml), endogenous level (+4 ng/ml), and endogenous level (+8 ng/ml) for human CSF assay] were prepared by serial dilution after 24(S)-HC working solution was spiked into blank biological matrix. The LLOQ samples for human plasma (1 ng/ml) and CSF (0.025 ng/ml) were prepared in 5% BSA and 2.5% HP-β-CD solutions, respectively. The 24(S)-HC in the DQC samples was higher than the upper limit of quantification (ULOQ) (200 ng/ml for human plasma; 5 ng/ml for human CSF). The human plasma and CSF DQC samples were diluted 1:4 with 5% BSA and 2.5% HP-β-CD solutions, respectively, prior to extraction.

### Sample preparation

For plasma, standards, quality controls (QCs), and blank or study samples (50 μl) were aliquoted into 10 ml glass test tubes. To each tube, internal standard working solution (50 μl) was added except that methanol-water (1:1) (50 μl) was used for a blank. The tubes were vortexed for approximately 15 s. To each tube was added 200 μl of acidic buffer [50 mM ammonium acetate, 1% formic acid (pH 3)] and 1 ml of methyl tert-butyl ether. The samples were vortexed for approximately 10 min and then centrifuged (approximately 2,200 rpm, 4°C, 5 min). The methyl tert-butyl ether phases (supernatants) were transferred to 1.2 ml glass inserts (VWR, West Chester, PA) after aqueous phases in samples were frozen in a dry-ice/ethanol bath. After methyl tert-butyl ether was evaporated with nitrogen at 35°C, to each insert was added 50 μl of derivitization reagent (a solution of 63 mg of *N*,*N*′-diisopropylcarbodiimide, 62 mg of nicotinic acid, and 61 mg of 4-(dimethylamino)pyridine in 5 ml of chloroform). The samples were heated at 50°C for 1 h, and the chloroform was removed with nitrogen at 35°C. The samples were reconstituted with 200 μl of methanol.

For CSF, standards, QCs, and blank or study samples (200 μl) were aliquoted into 2 ml glass test tubes. To each tube, internal standard working solution (50 μl) was added except that methanol-water (1:1) (50 μl) was used for a blank. The tubes were vortexed for approximately 15 s. To each tube was added 1 ml of methyl tert-butyl ether. The samples were vortexed for approximately 10 min and then centrifuged (approximately 2,200 rpm, 4°C, 5 min). The methyl tert-butyl ether phases (supernatants) were transferred to 1.2 ml glass inserts (VWR) after aqueous phases in samples were frozen in a dry-ice/ethanol bath. After methyl tert-butyl ether was evaporated with nitrogen at 35°C, to each insert was added 50 μl of derivitization reagent (a solution of 63 mg of *N*,*N*′-diisopropylcarbodiimide, 62 mg of nicotinic acid, and 61 mg of 4-(dimethylamino)pyridine in 5 ml of chloroform). The samples were heated at 50°C for 1 h, and the chloroform was removed with nitrogen at 35°C. The samples were reconstituted with 200 μl of methanol.

### LC-MS/MS analysis

LC-MS/MS analysis was conducted on a Shimadzu (Columbia, MD) Prominence HPLC system coupled with an Applied Biosystems/MDS Sciex (Ontario, Canada) 4000QTRAP mass spectrometer using multiple reaction monitoring (MRM). The HPLC system consisted of a Prominence HPLC system with a CBM-20A system controller, 4 LC-20AD pumps, a SIL-20ACHT autosampler, and a DGU-20A5R degasser.

The chromatography was performed using a C18 guard column (4 × 3.0 mm, Phenomenex) as the first dimension at ambient temperature and Eclipse XDB-C18 (3 × 100 mm, 3.5 μm; Agilent, Santa Clara, CA) as the second dimension at 50°C. The compartment of the autosampler was set at 4°C. Supplementary Fig. 1 is a schematic of the column and switching valve arrangement for 2D-LC. For the first dimension LC, mobile phase A (0.1% formic acid in water) and mobile phase B [0.1% formic acid in isopropanol-acetonitrile (1:2)] were operated with a gradient elution as follows: 0–0.6 min 60% B, 0.6–0.7 min 60–100% B, 0.7–5.5 min 100% B, 5.5–5.6 min 100–60% B, and 5.6–7.5 min 60% B at a flow rate of 0.6 ml/min. The solvent gradient for second dimension LC using 0.1% formic acid in water (phase C) and 0.1% formic acid in acetonitrile-methanol (1:4) (phase D) at a flow rate of 0.60 ml/min was as follows: 0–0.9 min 95% D, 0.9–6.0 min 95–100% D, 6.0–6.9 min 100% D, 6.9–7.0 min 100–95% D, and 7.0–7.5 min 95% D. Valve 1 was kept at the A position during 0–0.6 min and 1.2–7.5 min, and at the B position during 0.6–1.2 min. Valve 2 was kept at the A position during 0–5.0 min and 7.0–7.5 min, and at the B position during 5.0–6.9 min. The injection volume was 5 and 10 μl for human plasma and CSF samples, respectively. The ESI source temperature was 600°C; the ESI needle was 5,000 V; the declustering potential was 50 V; the entrance potential was 10 V; and the collision cell exit potential was 10 V. The collision and curtain gas were set at medium and 20, respectively. Both desolvation gas and nebulizing gas were set at 45 l/min. For MRM, the collision energies for mass transitions of *m/z* 307.2–124.0 [quantifier for 24(S)-HC], *m/z* 307.2–490.4 [qualifier for 24(S)-HC], and *m/z* 310.7–124.0 (D7-24-HC, internal standard) were 23, 13, and 23 V, respectively. The dwell time was set at 50 ms for each mass transition. Data were acquired and analyzed by Analyst software (version 1.5.1). Calibration curves were constructed by plotting the corresponding peak area ratios of analyte/internal standard versus the corresponding analyte concentrations using weighted (1/x^2^) least-squares regression analysis.

### Linearity, precision, and accuracy

The linearity response of analytes was assessed over their respective calibration range from three batches of analytical runs. The precision and accuracy of the assay were determined for each analyte at LLOQ, LQC, MQC, and HQC concentration levels in human plasma and CSF over the three batch runs. The DQC was used to assess the dilution integration. These QC concentrations included the known fortified levels added to the plasma or CSF plus the endogenous concentration of analyte. For each QC concentration, analysis was performed in six replicates on each day, except for DQCs for which three replicates were prepared. Precision and accuracy are denoted by percent coefficient of variance (CV) and percent relative error (RE), respectively. The accuracy and precision were required to be within ±15% RE of the nominal concentration and ≤15% CV, respectively, for LQC, MQC, HQC, and DQC samples. The accuracy and precision were required to be within ±20% RE of the nominal concentration and ≤20% CV for LLOQ samples in the intra-batch and inter-batch assays (39).

### Sample stability

For 24(S)-HC, long-term storage, freeze/thaw stabilities, and stabilities on the bench-top and in the autosampler were determined at the LQC and HQC concentration levels (n = 3). Long-term storage stability of analyte in human plasma and CSF was tested up to 48 and 34 days upon storage at −80°C, respectively. Bench-top stability was evaluated from human plasma and CSF that were kept on the lab bench at room temperature for 4 h before sample extraction. Freeze/thaw stability was tested by freezing the samples overnight, followed by thawing to room temperature the next day. This process was repeated three times. In the autosampler, stability was tested over three days by injecting the first batch of the validation samples. Stock solution stability was established by quantification of samples from dilution of two stock solutions that had been stored at −20°C for 48 days and at room temperature on the bench for 18 h, respectively, to the final solution (200 ng/ml in methanol). A fresh standard curve was established each time.

### Analysis of clinical samples

Samples consisting of calibration standards in duplicate, a blank, a blank with internal standard, QCs (LQC, MQC, and HQC), and unknown clinical samples were analyzed. The standard curve covered the expected unknown sample concentration range, and samples that exceeded the highest standard could be diluted and re-assayed. In the dilution sample re-assay, a DQC in triplicate was also included in the analytical run. The results of the QC samples provided the basis for accepting or rejecting the run according to Food and Drug Administration guidelines (39).

### Drug administration and sample collection in pharmacodynamic study

This clinical study was approved by the Institutional Review Board of the Eunice Kennedy Shriver National Institute of Child Health and Human Development. Permission from guardians and assent, when possible, were obtained from all participants. The study was posted on ClinicalTrials.gov (NCT01747135) and use of HP-β-CD was covered under IND 113273. Three NPC1 subjects were admitted to the NIH Clinical Center Intensive Care Unit and an Ommaya reservoir was surgically placed on the nondominant side. The 50 mg HP-β-CD dose was prepared in 5 ml of an isotonic salt solution. Vehicle (saline) and HP-β-CD doses were administered intracerebroventricularly via the Ommaya reservoir. CSF and blood, 1 and 2 ml respectively, were collected at predose, 0.25, 0.5, 1, 3, 8, 24, 36, 48, and 72 h postdose. Blood samples were collected in K_2_EDTA tubes and the plasma fraction was separated within 1 h by centrifugation at 1,500 *g* for 10 min at 4–8°C. The CSF samples were collected in tubes containing 25 mg of HP-β-CD. Plasma and CSF samples were kept at −80°C before analysis. The area under the curve (AUC) was used to assess pharmacodynamic responses of 24(S)-HC to HP-β-CD and the deviation of a signal from its baseline value (40). The AUC was calculated by the linear trapezoidal method using GraphPad Prism software (version 6.0) (GraphPad Software, La Jolla, CA).

## RESULTS

### LC-MS/MS assay development

**Fig. 1.**
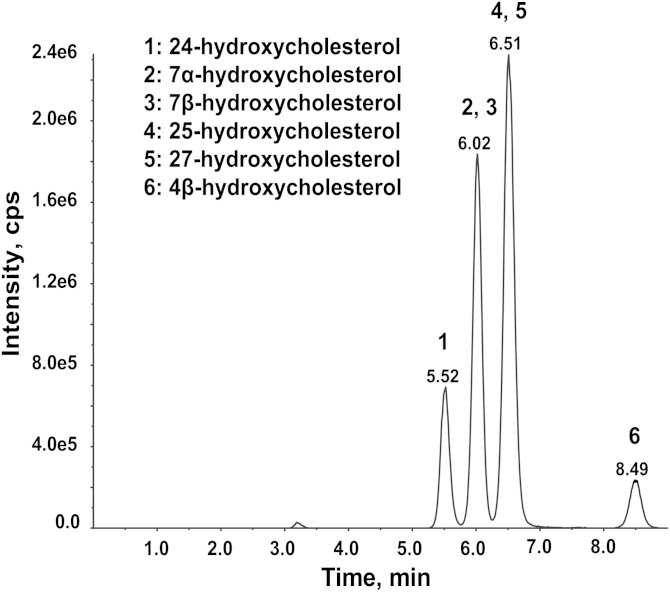
Separation of 24(S)-HC nicotinate (1) from nicotinates of 7α-hydroxycholesterol (2), 7β-hydroxycholesterol (3), 25-hydroxycholesterol (4), 27-hydroxycholesterol (5), and 4β-hydroxycholesterol (6) present in the human plasma on an Eclipse XBD column (3 × 100 mm, 3.5 μm) using 0.1% formic acid in water (phase A) and 0.1% formic acid in acetonitrile/methanol (1:4) (phase B) at a flow rate of 0.60 ml/min. The gradient was as follows: 0–5.0 min 95–100% B, 6.0–6.9 min 100% B, 6.9–7.0 min 100–95% B, and 7.0–9 min 95% B.

Although underivatized, 24(S)-HC in plasma can be detected by ESI as [M+NH_4_]^+^ on a 4000QTRAP and by APCI as [M+H-H_2_O]^+^ on Thermo TSQ triple-quadrupole mass spectrometers, the sensitivities are insufficient to detect unesterified 24(S)-HC in CSF. Derivatization of 24(S)-HC to its picolinyl ester significantly increased the detection sensitivity; however, a long LC run (>20 min) was necessary to separate the 24(S)-HC from other isomers (35). By contrast, we found that the 24(S)-HC nicotinate derivative was easily separated from other major isomers (7α-hydroxycholesterol, 7β-hydroxycholesterol, 25-hydroxycholesterol, 27-hydroxycholesterol, and 4β-hydroxycholesterol) present in the human plasma using an Eclipse XBD column (3 × 100 mm, 3.5 μm) with a 9 min LC run time including equilibration time (**Fig. 1**).

**Fig. 2.**
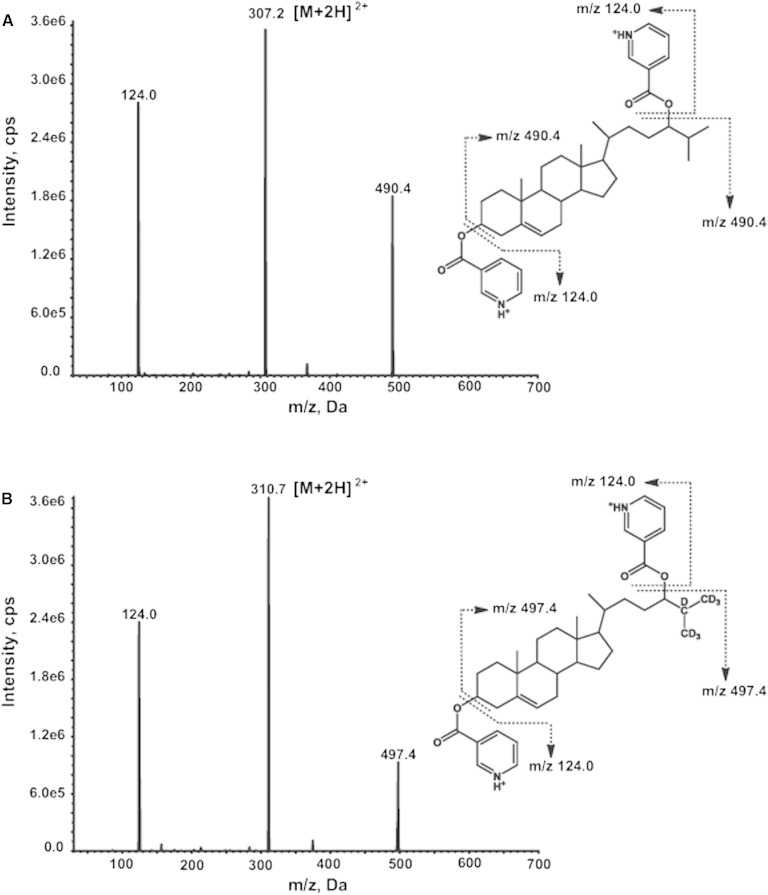
Product ion spectra and proposed fragmentation pathways of nicotinates of 24(S)-HC (A) and D7-24-HC (B).

Unlike the picolinyl ester derivative of 24(S)-HC of which the major pseudo-molecular ion is [M+Na]^+^, the nicotinate derivative exhibits abundant protonated molecular ions, [M+H]^+^ and [M+2H]^2+^ at *m/z* 613.4 and 307.2, respectively. Both [M+H]^+^ and [M+2H]^2+^ ions generate major product ions at *m/z* 124 and 490.4, respectively. The *m/z* 124.0 ion is a protonated nicotinic acid, and the *m/z* 490.4 ion is generated by neutral loss of a nicotinic acid from the [M+H]^+^ ion or elimination of a protonated nicotinic acid from the [M+2H]^2+^ ion. The MRM transition *m/z* 307.7 → 124.0 showed higher sensitivity than other transitions (*m/z* 613.4 → 124.0, *m/z* 307.7 → 490.4, and *m/z* 613.4 → 490.4) and therefore was chosen as quantifier for 24(S)-HC. The MRM transition *m/z* 307.2 → 490.4 was used as qualifier. Similarly, the MRM transition *m/z* 310.7 → 124.0 was chosen for monitoring of D7-24-HC (internal standard). The product ion spectra and proposed fragmentation pathways of [M+2H]^2+^ ions of nicotinates of 24(S)-HC and D7-24-HC are given in **Fig. 2**.

To increase the throughput, the derivitization mixture was directly injected into the Eclipse XBD column. However, the retention time of 24(S)-HC nicotinate derivative changed over time when a large number of samples were injected, likely due to blockage of active sites of stationary phase by the matrix buildup. To minimize matrix interference, a 2D chromatography approach was employed. A C18 guard column (4 × 3.0 mm, Phenomenex) was set as the first-dimensional column and a 0.6 min isocratic elution with 60% mobile phase B was used to remove excess derivatization reagents and by-products. The 24(S)-HC nicotinate derivative was switched and transferred to the second-dimensional column (Eclipse XBD column, 100 × 3 mm i.d., 3.5 μm) by elution with 95% mobile phase D for a brief period from 0.7 to 1.2 min of retention time through the first six-port switching valve. After the 24(S)-HC nicotinate derivative was transferred to the second-dimensional column, the first six-port switching valve was switched back to the original position, and the first-dimensional column was washed with 100% mobile phase B for 4.8 min followed by equilibration with 60% mobile phase B for 2 min. A gradient from 95 to 100% mobile phase D starting from 0.9 to 6 min of retention time was used for the second-dimensional column to elute 24(S)-HC nicotinate derivative. Then the second-dimensional column was washed with 100% mobile phase D for 0.9 min and equilibrated with 95% mobile phase D for 0.5 min. The acquisition window on the mass spectrometer was set for 2 min via a second two-position switching valve so that the mass spectrometer ion source was kept clean and well maintained. The overall run time was 7.5 min per sample. Therefore, 2D-LC-MS/MS removed most matrix interference and late eluted 4β-hydroxycholesterol on the first-dimensional column, improved the robustness of the second-dimensional column, and shortened the LC run time to 7.5 min.

Earlier methods to extract 24(S)-HC from plasma used a mixture of chloroform and methanol to simultaneously disrupt lipoproteins and partition 24(S)-HC into chloroform. We found that the acidification of plasma with ammonium acetate-formic acid buffer (pH 3) affected the protein conformation, which in turn led to reduced affinity between 24(S)-HC and plasma proteins. We further used a liquid-liquid extraction with methyl-tert-butyl ether to extract 24(S)-HC from plasma and CSF. An advantage of liquid-liquid extraction with methyl-tert-butyl ether is its high efficiency in removing the phospholipids that are the major source of matrix effect (ionization suppression or enhancement) (41).

### Overcoming nonspecific adsorption issues for 24(S)-HC in human CSF samples

The lack of a significant amount of protein and lipids, as well as the relatively high ionic strength in CSF samples, can be associated with the loss of lipophilic and highly protein bound molecules via nonspecific binding or adsorption to the hydrophobic surface of the polypropylene container in which they are collected, stored, or processed. Failure to adequately address this issue would result in underestimated CSF analyte concentrations, as compounds with log D larger than 3.8 would be expected to experience ≥20% adsorption loss in untreated CSF (42). The log D of 24(S)-HC is 7.56 (43), suggesting that it is very likely to be lost by nonspecific binding to a polypropylene container. To confirm this prediction, we conducted a nonspecific binding diagnostic experiment which consisted of five consecutive transfer and incubation steps of CSF samples in polypropylene tubes (44), and found that 55% of 24(S)-HC was lost after five consecutive transfers and incubations. To prevent nonspecific binding of 24(S)-HC in CSF samples to the polypropylene container, HP-β-CD was added to a final concentration of 2.5% and the recovery of 24(S)-HC was greater than 93.1% after five consecutive transfers and incubations. Based on these findings, the clinical samples were collected in polypropylene tubes preloaded with HP-β-CD.

### Selection of surrogate matrix for standard curves

As no 24(S)-HC-free human plasma and CSF are available, we prepared calibration standards by spiking the analyte in surrogate matrixes. Five percent BSA in water was used to simulate generic binding of the analyte to endogenous proteins, and served as the surrogate standard curve matrix for plasma samples. HP-β-CD (2.5%) in water was used as the surrogate standard curve matrix for CSF samples, because 2.5% HP-β-CD was used to prevent nonspecific binding of 24(S)-HC in CSF. Because surrogate matrixes were used, the impact of matrixes was investigated using another set of standard curves prepared in pooled human plasma and CSF. The standard curves prepared in human plasma and CSF were parallel to those prepared in 5% BSA and 2.5% HP-β-CD, respectively, and the differences in slopes of the standard curves in surrogate and authentic matrixes were 0 and 3.1% for plasma and CSF, respectively. The intercepts of the surrogate matrix standard curve were close to zero, while they were slightly greater than zero for the authentic matrixes due to the presence of the endogenous analytes (supplementary Table 1). These results suggested that the same responsiveness of 24(S)-HC in different matrixes was observed and calibration curves prepared in surrogate matrixes were suitable for analysis of plasma and CSF samples.

### Extraction efficiency and matrix effects

To evaluate the recoveries of the 24(S)-HC from human plasma, CSF and surrogate standard curve matrixes (5% BSA and 2.5% HP-β-CD), signals of D7-24-HC from preextraction spiked samples were compared with those of postextraction spiked samples. The recoveries of 24(S)-HC were 105, 91, 91, and 93% for plasma, 5% BSA, CSF, and 2.5% HP-β-CD, respectively. The matrix factors (45) for 24(S)-HC were assessed by comparing the peak response of the D7-24-HC from postextraction spiked samples to equivalent pure compound solutions in methanol. Matrix factors were 1.04, 0.988, 0.938, and 1.01 for plasma, 5% BSA, CSF, and 2.5% HP-β-CD, respectively, suggesting that there are no significant matrix effects for 24(S)-HC in these matrixes.

### Selectivity

**Fig. 3.**
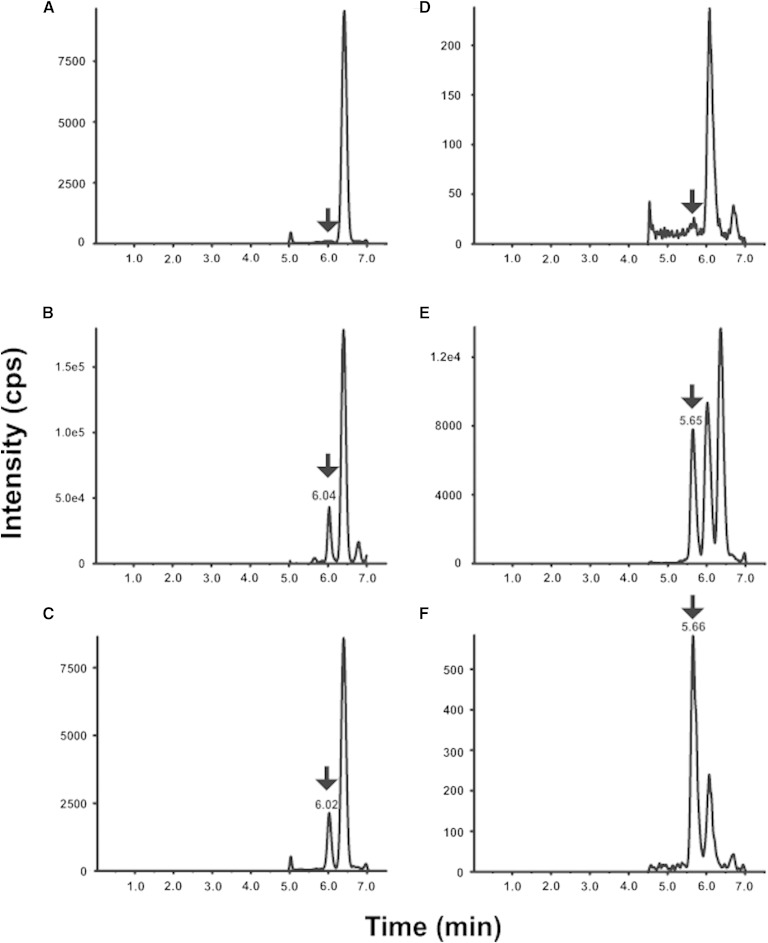
2D-LC-MS/MS chromatograms for 24(S)-HC in blank for plasma (A) and CSF (D), human plasma (B) and CSF (E), and LLOQ for plasma (C) and CSF (F). The 24(S)-HC peak position is marked by an arrow ↓. The retention time of 24(S)-HC in plasma and CSF differs slightly due to the different lot of columns used.

To ascertain the selectivity of the plasma and CSF methods, blank (5% BSA solution for plasma method and 2.5% HP-beta-CD solution for CSF method) with and without internal standard and six independent human plasmas were analyzed. As shown in **Fig. 3A, D**, no interfering peaks to analyte and internal standard from blanks for plasma and CSF were observed. There are no interfering peaks to analyte from blank with internal standard for plasma and CSF. In the highest calibrator (ULOQ, 200 ng/ml for human plasma; 5 ng/ml for human CSF) without internal standards, there are no interfering peaks to internal standard.

All the human plasma (Fig. 3B) and CSF (Fig. 3E) used for preparation of the standard curves contained endogenous levels of 24(S)-HC. There were no interfering peaks from the human plasma and CSF at the retention time and in the MRM channel of the internal standard.

The selectivity was also evaluated by comparison of the branching ratio (46–48) of the mass transitions from six individual blank plasma and six individual blank CSF samples with that of the highest calibrator (ULOQ sample) prepared in methanol. The branching ratio is the ratio of peak areas of two mass transitions of 24(S)-HC (*m/z* 307.2 → 124.0 and *m/z* 307.2 → 490.4), used to assure specificity of the detection. The selectivity was further confirmed by the branching ratio of six individual blank plasma and six individual blank CSF samples within 10% of the value of the highest calibrator (supplementary Table 2).

### Sensitivity

The LLOQ for plasma and CSF were prepared in BSA and 2.5% HP-β-CD solutions and at 1 and 0.025 ng/ml, respectively. The LLOQ samples were processed and analyzed with a calibration curve and QC samples. The intra-run precisions at LLOQ level were 2.3–13.3% CV and 3.5–6.3% CV for plasma and CSF, respectively. The intra-run accuracy levels were 1.3 to 6.7% RE and −0.7 to 13.3% RE for plasma and CSF, respectively. The inter-run precision was 7.6 and 7.9% CV for plasma and CSF, respectively. The inter-run accuracy was 4.6 and 4.1% RE for plasma and CSF, respectively (**Table 1**). A typical MRM chromatogram at the LLOQ concentration is shown in Fig. 3C, F.

### Accuracy and precision

The accuracy and precision of the method were assessed by analyzing QC samples along with a calibration curve on three different days. The calibration curve consisted of eight standards of different concentrations, each in duplicate, ranging from 1 to 200 ng/ml for plasma and 0.025 to 5 ng/ml for CSF. The calibration curve [24(S)-HC peak area/internal standard peak area for y-axis and analyte concentration for x-axis] of 24(S)-HC was obtained using the least-squares linear regression fit (y = ax + b) and a weighting factor of 1/x^2^. Excellent results were obtained for the calibration curves, as the deviations of the back-calculated concentrations from their nominal values were within 15% for all the calibration standards in the 3 days of validation. The coefficients of determination (r^2^) greater than 0.99 were observed for the calibration curves. All the QC samples were prepared in human plasma or CSF, and the endogenous levels of 24(S)-HC were determined by mean of multiple replicates (n = 12). The endogenous levels were used to calculate the nominal concentrations of the spiked MQC, HQC, and DQC. The plasma and CSF DQC were diluted five times with 0.5% BSA and 2.5% HP-β-CD solutions before extraction, respectively, and followed the procedure for other samples. The results of the QC samples in the three validation runs and dilution integration are shown in Table 1. The analysis of the plasma- and CSF-based QC samples demonstrated acceptable precision and accuracy based on the preset validation criteria of ±15% CV and 15% RE.

**TABLE 1.** Accuracy and precision of QC samples

Analytical Batch Number	Matrix	Human Plasma					Human CSF				
	QC Level	LLOQ	LQC	MQC	HQC	DQC	LLOQ	LQC	MQC	HQC	DQC
	Nominal Concentration	1 ng/ml	8.64 ng/ml	83.6 ng/ml	159 ng/ml	309 ng/ml	0.025 ng/ml	0.244 ng/ml	2.24 ng/ml	4.24 ng/ml	8.24 ng/ml
1	Intra-run Mean	1.06	8.92	87.3	160	337	0.0249	0.254	2.36	4.49	9.18
	Intra-run CV (%)	2.3	4.4	5.2	4	4.8	6.3	2.3	2.2	2.8	2.2
	Intra-run RE (%)	5.8	3.3	4.3	1	9.2	−0.4	4.1	5.2	5.9	11.4
	n	6	6	6	6	3	6	6	6	6	3
2	Intra-run Mean	1.01	8.51	89	164	—	0.0283	0.251	2.41	4.62	—
	Intra-run CV (%)	13.3	3.5	5.2	2.5	—	3.5	4.1	1.5	1.2	—
	Intra-run RE (%)	1.3	−1.5	6.4	3.3	—	13.3	2.8	7.4	9	—
	n	6	6	6	6	—	6	6	6	6	—
3	Intra-run Mean	1.07	8.56	85.4	164	—	0.0248	0.236	2.33	4.28	—
	Intra-run CV (%)	2.4	2.7	5.6	1.8	—	4.6	3.9	1.5	3.5	—
	Intra-run RE (%)	6.7	−1	2.1	3.5	—	−0.7	−3.1	4.2	0.9	—
	n	6	6	6	6	—	6	6	6	6	—
Inter-batch	Inter-run Mean	1.05	8.64	87.2	163	—	0.026	0.247	2.37	4.46	—
	Inter-run CV (%)	7.6	3.9	5.3	2.9	—	7.9	4.6	2.1	4.1	—
	Inter-run RE (%)	4.6	0	4.3	2.6	—	4.1	1.3	5.6	5.3	—
	n	18	18	18	18	—	18	18	18	18	—

### Carryover

To evaluate carryover, a blank sample was immediately injected following the highest standard (200 ng/ml for plasma and 5 ng/ml for CSF). No carryover was observed in the regions of interest.

### Stability

Stability of the 24(S)-HC in the human plasma and CSF was evaluated under a variety of conditions to establish length of storage and sample processing conditions. The bench-top stability study showed that the 24(S)-HC was stable in human plasma and CSF for 4 h at room temperature. The stability of 24(S)-HC was determined to be acceptable in human plasma and CSF following three freeze-thaw cycles. For processed samples (autosampler stability), the 24(S)-HC nicotinate was stable for 3 days at 4°C. The 24(S)-HC was determined to be stable for 48 and 34 days at −80°C in human plasma and CSF, respectively.

The 24(S)-HC in standard curve matrixes and stock solution were stable for 18 h at room temperature and for 48 days at −80°C.

### Evaluation of 24(S)-HC as a biomarker in phase 1 trial of HP-β-CD in NPC1 patients

NPC1 is a fatal neurodegenerative lysosomal storage disorder characterized by abnormal accumulation of unesterified cholesterol and sphingolipids in late endosomes/lysosomes of many cell types (49). HP-β-CD has been shown to prevent neurodegeneration and prolong survival in NPC1 animal models (50–52) and is currently being studied in a phase 1 trial at NIH (38). Administration of HP-β-CD in the animal models promotes a rapid redistribution of sequestered cholesterol from the late endosomes/lysosomes to the endoplasmic reticulum, where the cholesterol is metabolized to 24(S)-HC in the CNS (53). Accordingly, CSF and plasma 24(S)-HC are expected to elevate upon treatment with HP-β-CD. The phase 1 trial for intracerebroventricular HP-β-CD was designed to primarily evaluate safety and pharmacokinetics of HP-β-CD (54), with exploratory efforts focused on pharmacodynamic activity. One aim of our studies was to determine whether 24(S)-HC in the plasma and CSF is a useful pharmacodynamic measurement of HP-β-CD-mediated amelioration of cholesterol storage in CNS.

**Fig. 4.**
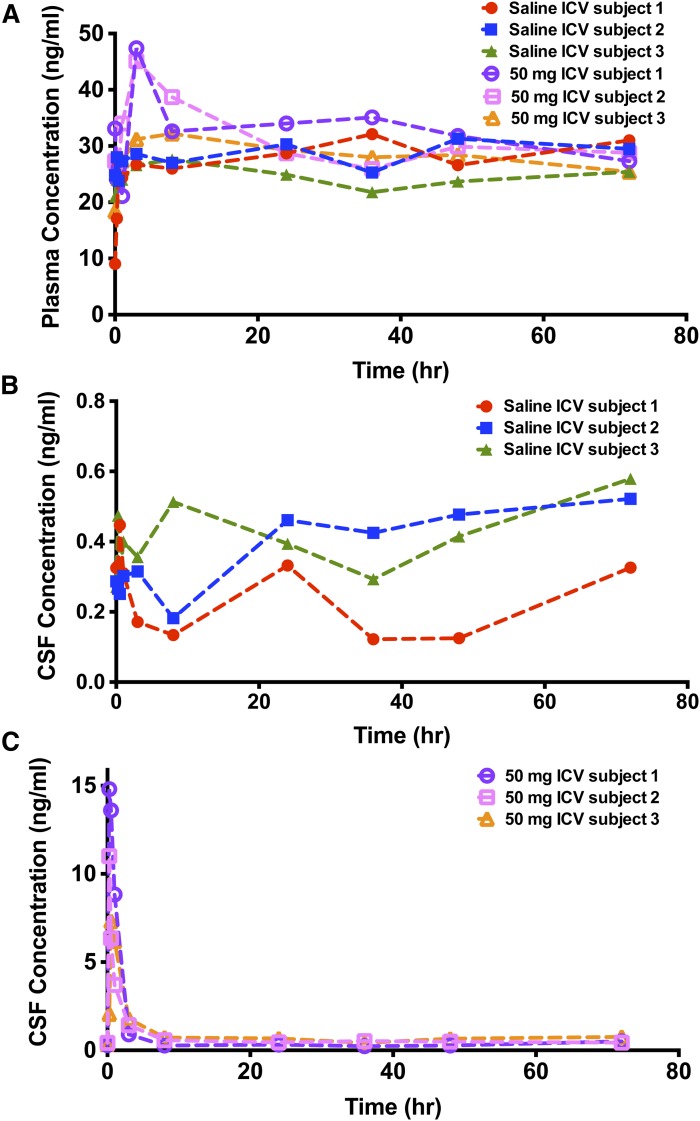
Concentration-time profile of 24(S)-HC in plasma (A) and CSF (B, C) obtained from a phase 1 study of HP-β-CD in NPC1 subjects administered saline and 50 mg HP-β-CD intracerebroventricularly.

The effects of vehicle or HP-β-CD (50 mg) intracerebroventricular administration on the time course of plasma and the CSF level of 24(S)-HC in three NPC1 subjects are shown in **Fig. 4**. The intracerebroventricular administration of HP-β-CD elicited a significant increase of 24(S)-HC from basal levels in the CSF. The C_max_ increased 21- to 50-fold of that of basal levels at 0.25–0.5 h post-dose, and the AUC of 24(S)-HC from 0 to 72 h post-HP-β-CD administration increased 1.6- to 2.9-fold of that of saline treatment values. The plasma 24(S)-HC also increased 1.43- to 1.75-fold of that of basal levels at 3–8 h following administration of HP-β-CD, and the C_max_ was delayed in plasma as compared with CSF. The AUC of 24(S)-HC in plasma following intracerebroventricular injection of HP-β-CD increased 6–16%, compared with saline treatment values.

## DISCUSSION

In the present study, the intended use of the assay was for measuring the 24(S)-HC as a pharmacodynamic biomarker in clinical trials, and the data generated to be used for critical decision-making in the development of HP-β-CD as new drug candidate. Thus, an increase in the rigor of 24(S)-HC method validation is essential. To ensure high quality data, a rigorous full validation was conducted to extensively evaluate the performance of the assay according to Food and Drug Administration guidelines (39) and “fit-for-purpose” strategy (55, 56). We developed a highly selective, sensitive, and high-throughput 2D-LC-MS/MS assay for quantification of 24(S)-HC in human plasma and CSF. This assay used a “surrogate matrix” strategy that reduced the issue of potential interference from the endogenous analyte. The assay demonstrated excellent accuracy, precision, linearity, and selectivity for the intended purpose of using plasma and CSF 24(S)-HC as biomarkers in clinical trials. The LLOQ was sufficient to capture the basal levels of 24(S)-HC in human plasma and CSF. Stability of the analytes was also thoroughly investigated in the current study, and it was found that 24(S)-HC in plasma and CSF demonstrated good bench-top stability, freeze-thaw stability, and long-term storage stability. In CSF, 24(S)-HC is subjected to absorption loss due to nonspecific binding to the polypropylene container, which is prevented by the presence of 2.5% HP-β-CD. Accordingly, appropriate CSF sample collection for clinical studies was established to prevent significant absorption loss. Finally, we demonstrated the utility of the validated 24(S)-HC assays in the context of a phase 1 clinical trial, in which CSF and plasma 24(S)-HC concentrations were employed as pharmacodynamic markers for determining HP-β-CD target engagement.

Although quantification of 24(S)-HC in biological samples has been reported using GC-MS or LC-MS/MS, none of the free 24(S)-HC assays previously reported were fully validated assays. Moreover, none of the previous assays addressed the absorption loss of 24(S)-HC in CSF via nonspecific binding to the polypropylene or polystyrene containers, which significantly limits the utility of these assays in clinical settings. Application of HP-β-CD to prevent absorption loss may be applied to analysis of other sterols and oxysterols in CSF. A recent paper by Bandaru and Haughey (28) reported an underivatized LC-MS/MS method for quantification of free 24(S)-HC in serum. A sensitive, but not selective, MRM transition [M+NH_4_]^+^ → [M+NH_4_-NH_3_-H_2_O]^+^ was used. The 24(S)-HC levels in serum are similar to our plasma data, suggesting that both methods can generate comparable results in control serum and some NPC1 plasmas. Compared with Bandaru and Haughey’s (28) method, our method offers several advantages. First, our method is more sensitive (LLOQ: 1 ng/ml vs. 10 ng/ml). Second, our method requires less sample volume (0.05 ml of plasma vs. 0.5 ml of serum). Third, our signal to noise ratio in biological sample (>20 in plasma vs. about 5 in serum) is higher, and run time (7.5 min vs. 12 min) shorter. Fourth, Bandaru and Haughey (28) reported that 24(S)-HC in sera from healthy subjects ranged from 4 to 21 ng/ml, indicating that 24(S)-HC in some samples (e.g., 4 ng/ml) cannot be reliably quantified, as they are below the LLOQ of the method. Thus, our method provides sufficient sensitivity to accurately measure these samples, and therefore may be more appropriate for pediatric studies, where sample volumes are limited.

Our method was developed on the 4000QTRAP, in which 24(S)-HC was derivatized with nicotinic acid to improve the detection sensitivity. Newer generations of mass spectrometers such as API5000, API5500, 5500QTRAP, and 6500QTRAP offer five to twenty times higher sensitivity than the 4000QTRAP. The underivatized method using nonselective MRM transition, as in Bandaru and Haughey’s (28) method on the newest 6500QTRAP, may achieve the same LLOQ as our method; however, the signal to noise ratio in the plasma/serum sample is still inferior to our method. Nonetheless, coupling 6500QTRAP with nicotinic acid derivatization may provide enough sensitivity to detect 24(S)-HC in dried blood spot samples (equivalent to 1.6 μl plasma/3 mm disk) that is ideal for pediatric studies in infants and children.

2D chromatography was employed in our assay. Although 2D chromatography involves more complex instrumentation and method, it has a number of advantages over separation with a single column. Combination of different separation mechanisms offers a high peak capacity to resolve samples of great complexity. The improved separation capacities reduce/eliminate the interferences, background noises, and ion suppression, all of which improve sensitivity. The peaks eluting before and after the window of the peak transfer from the first to the second column are directed to waste, thus there is less potential for matrix buildup on the second analytical column. The analytical column has a longer life, and consequently the method is more robust. The gradient run time can be reduced by equilibration of a column during the elution on another column. In this study, we used 2D chromatography to achieve online clean up, prolong column life, increase method robustness, and reduce the run time. Although only one switching valve is required to perform 2D chromatography, we used a second switching valve to keep the ion source clean. Using this method, we have analyzed 1,700 samples from the phase 1 clinical trial on a single analytical column, without needing to clean the mass spectrometer ion source. Advances in the theory of 2D separations (57, 58), instrument technology (59), and control software (60) in recent years have led to widespread applications in proteomics (60, 61), metabolomics (62), and pharmaceutical analysis (63). To date, adoption of 2D chromatography for the separation of lipids has been slow (64), but it is anticipated that we will see more application of this powerful tool to lipid research in the near future.

24(S)-HC has been evaluated as a pharmacodynamic biomarker for HP-β-CD treatment of NPC1 animal models (53). Subcutaneous administration of HP-β-CD has been shown previously to delay neurodegeneration and to prolong lifespan in NPC1 mice (50–52). We observed significant increases of 24(S)-HC in NPC1 mouse plasma after subcutaneous administration of 4,000 mg/kg or stereotactic injection of 6 mg/kg of HP-β-CD, and in NPC1 cat plasma and CSF after intracisternal administration of 30 and 120 mg (53). Although a subsequent report suggested that intraperitoneal injection of 4,000 mg/kg of HP-β-CD to NPC1 mice had no effect on 24(S)-HC in plasma (65), this conclusion was based on quantification of 24(S)-HC using an ELISA kit, which is unreliable because of the vast excess of cross reactant cholesterol [cross reactivity: 0.004% (66), 1.23–2.41 mg/ml (67)] that is 59,000-fold more abundant than 24(S)-HC [30 ng/ml (4)] and the interference contribution from cholesterol is about 2.3-fold that of 24(S)-HC. In the present study, we used the fully validated LC-MS/MS assays to measure 24(S)-HC in plasma and CSF from human NPC1 subjects enrolled in the NIH-sponsored phase 1 trial of intracerebroventricular administration of HP-β-CD. Our results showed that 24(S)-HC concentrations were significantly increased in the plasma and CSF, indicative of a biochemical response to the HP-β-CD treatment, thus confirming our findings in animal studies.

### Note added in proof

The original accepted version of this manuscript had the third author listed as “Nicole Yanjanin.” The authors advised the *Journal* after acceptance that the third author’s name should be listed as “Nicole Y. Farhat.” This correction has been made in this final published version of the manuscript.

## Supplementary Material

Supplemental Data
