# Supplementary material for: A validated LC-MS/MS assay for quantification of 24(S)-hydroxycholesterol in plasma and cerebrospinal fluid
Source: J Lipid Res. 2015 Jun;56(6):1222–33. doi: 10.1194/jlr.D058487 (PMC4442878; doi:10.1194/jlr.D058487)
Supplement: Supplemental Data [file supp_56_6_1222__index.html]

A Validated LC-MS/MS Assay for Quantification of 24(S)-Hydroxycholesterol in Plasma and Cerebrospinal Fluid — A Validated LC-MS/MS Assay for Quantification of 24(S)-Hydroxycholesterol in Plasma and Cerebrospinal Fluid — A validated LC-MS/MS assay for quantification of 24(S)-hydroxycholesterol in plasma and cerebrospinal fluid — Supplemental Data 

# A validated LC-MS/MS assay for quantification of 24(S)-hydroxycholesterol in plasma and cerebrospinal fluid

## Supplemental Data

**Files in this Data Supplement:**

- Supplementary Table S1-2 and Figure S1\_revised1 - Supplementary Table S1. Parameters of standard curves prepared in surrogate and authentic matrixes Supplementary Table S2. Branching ratio of plasma and highest standard (ULOQ) in methanol Supplementary Figure S1. Schematic representation of the 2D-LC?MS/MS system.
