# Supplementary material for: A validated LC-MS/MS assay for quantification of 24(S)-hydroxycholesterol in plasma and cerebrospinal fluid
Source: J Lipid Res. 2015 Jun;56(6):1222–33. doi: 10.1194/jlr.D058487 (PMC4442878; doi:10.1194/jlr.D058487)
Supplement: Supplemental Data [file supp_D058487_jlr.D058487-1.pdf]

Supplementary Table S1. Parameters of standard curves prepared in surrogate and authentic matrixes

Matrix	Slope	Intercept	R
5% BSA (Surrogate)	0.145	0.000163	0.9978
Plasma (Authentic)	0.145	0.125	0.9991
2.5% HP-β-CD (Surrogate)	0.428	0.00168	0.9978
CSF (Authentic)	0.456	0.116	0.9963

Supplementary Table S2. Branching ratio of plasma and highest standard (ULOQ) in methanol

Matrix	Sample	Branching ratio	% Difference from ULOQ
Plasma	Plasma-1	24.33	-5.8
	Plasma-2	24.57	-4.9
	Plasma-3	23.81	-7.9
	Plasma-4	25.22	-2.4
	Plasma-5	26.15	1.2
	Plasma-6	27.56	6.7
	ULOQ of plasma	25.84	0
CSF	CSF-1	23.7	-7.9
	CSF-2	24.43	-5.1
	CSF-3	24.92	-3.2
	CSF-4	27.15	5.5
	CSF-5	24.23	-5.9
	CSF-6	25.91	0.7
	ULOQ of CSF	25.74	0

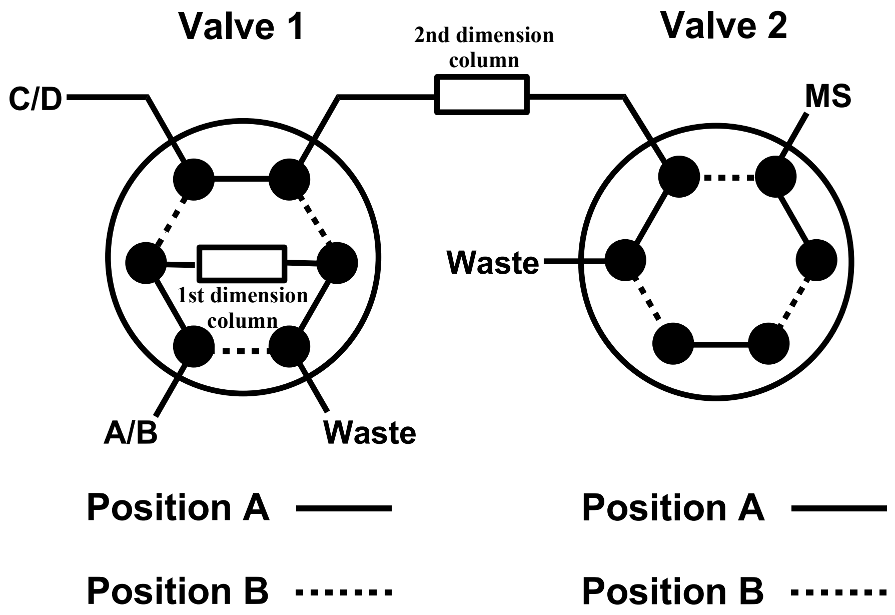

Supplementary Figure S1. Schematic representation of the 2D-LC–MS/MS system.
